# A methylene blue-based near-infrared fluorescent probe for rapid detection of hypochlorite in tap water and living cells†

**DOI:** 10.1039/c8ra01037d

**Published:** 2018-04-18

**Authors:** Xin Huang, Zhipeng Li, Tingting Cao, Qian Cai, Chengchu Zeng, Hua Fu, Liming Hu

**Affiliations:** College of Life Science and Bioengineering, Beijing Key Laboratory of Environmental and Oncology, Beijing University of Technology Beijing 100124 China huliming@bjut.edu.cn +86 10 67392001 +86 10 67396211; Key Laboratory of Bioorganic Phosphorus Chemistry and Chemical Biology (Ministry of Education), Tsinghua University Beijing 100084 China

## Abstract

A methylene blue-based near-infrared fluorescent probe was designed for the selective determination of hypochlorite (ClO^−^), over other reactive oxygen species or interfering agents. Acetylated methylene blue was synthesized by introducing the acetyl group into the methylene blue framework, which can specifically recognize exogenous and endogenous ClO^−^. The acetylated methylene blue fluorescent probe was characterized by ^1^H NMR, ^13^C NMR and HRMS. The response process and possible mechanism were studied using products of the probe. The emission response of the probe to ClO^−^ presented good linear relationship in the 0–60 μM concentration range, with the detection limit of 0.1 μM (measured at 660 nm and 690 nm). The absorption and emission wavelengths of acetylated methylene blue are both in the near-infrared region; in addition, the probe itself and the degradation products were well-dissolved in water and have almost no toxicity. The probe was used for intracellular ClO^−^ imaging and showed a large fluorescence enhancement (about 200-fold increase).

## Abstract 2

We developed a MB-based probe to detect OCl^−^, whose product is almost non-toxic. The fluorescence enhancement times are large.

## Graphical abstract

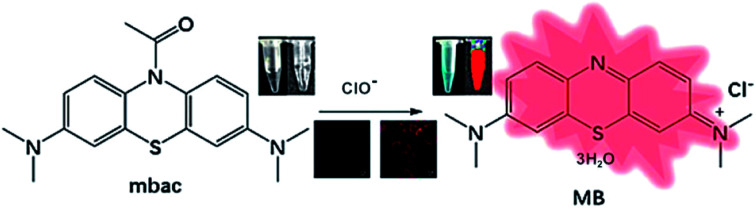

## Introduction

Hypochlorite (ClO^−^) is widely used as a disinfectant and bleaching agent daily, and it is one of the biologically important reactive oxygen species (ROS).^1^ ROS play important roles in the human immune defense system and the destruction of invading bacteria and pathogens.^2–4^

In living organisms, ClO^−^ is generated from hydrogen peroxide and chloride ion in activated neutrophils, catalyzed by myeloperoxidase (MPO).^1,5,6^ ClO^−^ is commonly used in disinfectants and bleaches.^7,8^ However, excessive formation of hypochlorite can also cause tissue damage and a series of human diseases, such as atherosclerosis, arthritis and even cancers, *etc.*^9,10^ Therefore, the rapid and sensitive detection of ClO^−^ is important in biological samples.

So far, many methods have been reported to detect ClO^−^, such as electrical analysis, potentiometric analysis, chemiluminescence, *etc.*^11^ Among them, the fluorescence method has the advantage of better sensitivity and selectivity.^12–14^ Fluorescent molecular probes can be employed as a powerful tool to track biomolecules in living systems due to their high sensitivity, real-time assay and non-invasive monitoring capability. In addition, the small-molecule fluorescent probe has many characteristics. The most attractive points are its low cost and low toxicity. Herein, a reactive fluorescent probe responsive toward ROS is designed.

Methylene blue (MB) is a photosensitizer approved by the FDA. This oxidized phenothiazine compound is widely used in clinical and basic research.^15^ Recently, MB was also applied as a NIR (near-infrared) imaging agent in image-guided surgery.^16^ As an ideal fluorescent group, the compound shows excellent photophysical properties, such as absorption and emission wavelength both in the NIR (more suitable for animal experiments to exclude background interference), almost no toxicity, and so on. So far, many new fluorescent probes have been reported based on near-infrared fluorophores.^17–23^

**Scheme 1:**
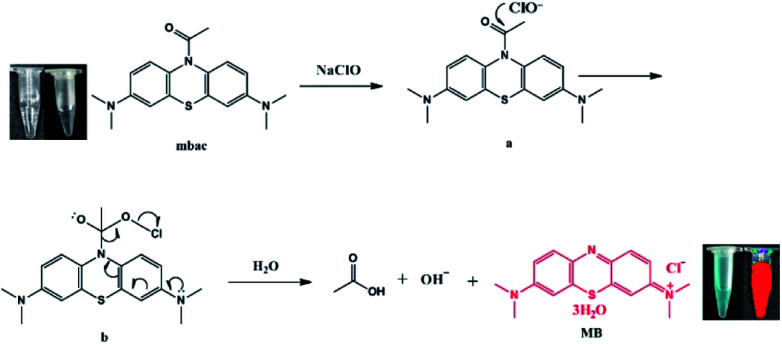
Possible mechanism: the cleavage of the amide bond of the fluorescent probe mbac results from interacting with ClO^−^. (The left side of the illustration is the view of the naked eye, and the right side is the view under the Pearl® Impulse Small Animal Imaging System).

Here, we developed a highly sensitive and selective hypochlorite turn-on sensor (MBAC, Scheme 1). The probe was synthesized by acetylation of methylene blue and characterized by ^1^H NMR, ^13^C NMR and HRMS. Upon reaction with ClO^−^, the acetyl group leaves mbac, thus altering the fluorescent properties of the probe and achieving the detection of ClO^−^. Thus, MBAC is almost non-fluorescent; however, strong near-infrared fluorescence was detected from the ClO^−^ response product. The advantage of mbac is that the emission wavelength is in the near-infrared region; especially, the fluorescence enhancement factor is large. The concentration is linearly related to the fluorescence intensity, and the detection limit is low.

We hypothesize that mbac amide bonds are cleaved by ClO^−^, resulting in the release of fluorescent group MB, while producing visible color changes and NIR emission (Scheme 1). This was verified by high-performance liquid chromatography (HPLC, Fig. 5). On the other hand, the experimental data show that MBAC was successfully utilized to detect endogenous hypochlorite in living cells, with excellent selectivity and sensitivity (the detection limit of 0.1 μM). According to its low cytotoxicity and high selectivity to ClO^−^, we see its application prospects and perform a series of tests on the probe.

## Experimental

### Materials

All reagents were purchased from commercial sources and used without further purification. MB and acetyl chloride were purchased from J&K Scientific Ltd. (Shanghai, China). Solutions of various testing species were respectively prepared from KCl, NaCl, LiCl, MgCl_2_, HgCl_2_, Cs_2_CO_3_, AgNO_3_, FeCl_2_, FeCl_3_, CaCl_2_, ZnCl_2_, CuCl_2_, CH_3_COONa, NaBr, NaI, Na_3_PO_4_, Na_2_HPO_4_, NaH_2_PO_4_, NaNO_2_, KMnO_4_, NaHCO_3_, NaF, Na_2_S_2_O_4_, GSH and NaClO. All solvents used in spectroscopic tests were spectroscopic grade. The water used in the experiments was deionized water.

### Methods

#### General procedure for synthesis of 1-[3,7-bis(dimethylamino)phenothiazin-10-yl]ethanone (mbac)

All syntheses were carried out at atmospheric pressure using magnetic agitation and with thin-layer chromatography (TLC) monitoring. MB (0.5 mmol, 187 mg), 1 mL water and 1.5 mL dichloromethane were mixed and stirred at 40 °C for 5 minutes. Then, sodium dithionite (1 mmol, 175 mg) and sodium bicarbonate (1 mmol, 84 mg) were added, stirring for 10 minutes.

**Scheme 2:**
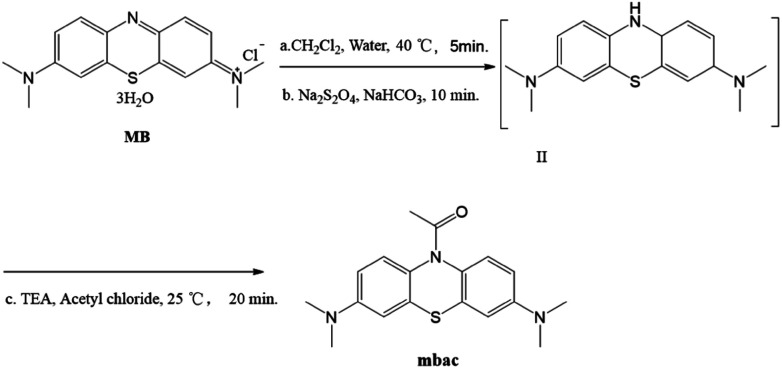
Synthesis of the probe mbac.

At this stage, the water phase was khaki-colored, and the organic phase was dark blue. The organic phase was separated and dried over anhydrous sodium sulfate, followed by the addition of triethylamine (0.6 mmol, 85 μL) to form a mixture. 0.6 mmol of acetyl chloride was added to 1 mL of dichloromethane and stirred. Then, the triethylamine mixture was slowly added dropwise to prevent the production of a large amount of white smoke, followed by stirring at room temperature for 20 minutes. The synthesis of a compound with similar structure has been reported.^24^ The reaction process is shown in Scheme 2. The product was purified by column chromatography (alumina, dichloromethane).

Mbac (122 mg, 74.4%), white powder. ^1^H NMR (400 MHz, CDCl_3_) *δ* (ppm) 2.17 (s, 3H, CH_3_), 2.94 (s, 12H, CH_3_), 6.62 (s, 2H, Ar–H), 6.71 (d, *J* = 2.4 Hz, 2H, Ar–H), 7.26 (s, 1H, Ar–H), 7.42 (s, 1H, Ar–H). ^13^C NMR (100 MHz, CDCl_3_) *δ* 170.54, 169.28, 147.94, 109.62, 109.83, 39.61, 23.92. HRMS (ESI): *m/z* calcd for C_18_H_21_N_3_OS [M + H]^+^ 328.1484, found 328.1452.

#### General procedure for reactive oxygen detection

The concentration of ClO^−^ was determined by standard titration method before use. The solutions of mbac (10 mM) were prepared in distilled deionized water. In the interference experiments, the probe was mixed in a 1 mL volume tube with various interfering agents. The solution of the test control group contains 500 μM ClO^−^, 10 μM probe. All experiments were performed at 25 °C for 25 minutes. 1 mL aliquots of the above-mentioned mixed solutions were pipetted into 1 cm cuvettes for spectral measurements. 5 nm bandpass filters were used for both excitation and emission wavelengths. An excitation wavelength of 660 nm was used for the acquisition of emission spectra.

#### Cell culture

LO2 cells and HepG2 cells used in this study were provided by Chinese PLA General Hospital (Beijing, China). HepG2 cells were previously established from human primary HCC. All samples were collected under the guidelines approved by the institutional review board of the Chinese PLA General Hospital. LO2 cells and HepG2 cells were grown on glass-bottom culture dishes (MatTek Co.) in DMEM supplemented with 10% (v/v) FBS and 1% (v/v) penicillin-streptomycin at 37 °C in a humidified 5% CO_2_ incubator. Before use, the adherent cells were washed three times with FBS free DMEM.

## Results and discussion

### Optimization of the experimental conditions

**Fig. 1:**
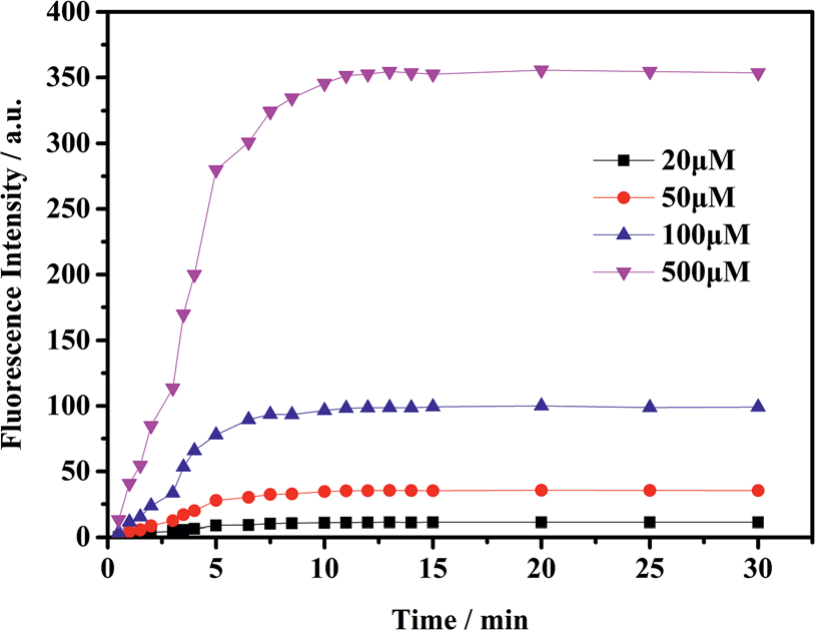
The time-dependent fluorescence changes (*λ*_ex_ = 660 nm) acquired from a mixture of the probe mbac (10 μM) and ClO^−^ (20 μM, 50 μM, 100 μM and 500 μM) in PBS (pH = 7.4) solution at room temperature. Data represent the average of three parallel experiments.

To check the response of the probe toward ClO^−^ at different time periods, temperature and pH levels, we conducted several sets of experiments. The fluorescence intensity of the probe toward ClO^−^ has been measured in different conditions. First, we measured the time-dependent emission curve (fluorescence intensity change) of the probe mbac. Fig. 1 shows that under different concentrations of ClO^−^, the trend of fluorescence intensity is consistent. Fluorescence intensity stopped increasing and stabilized at the highest value after 15 minutes.

**Fig. 2:**
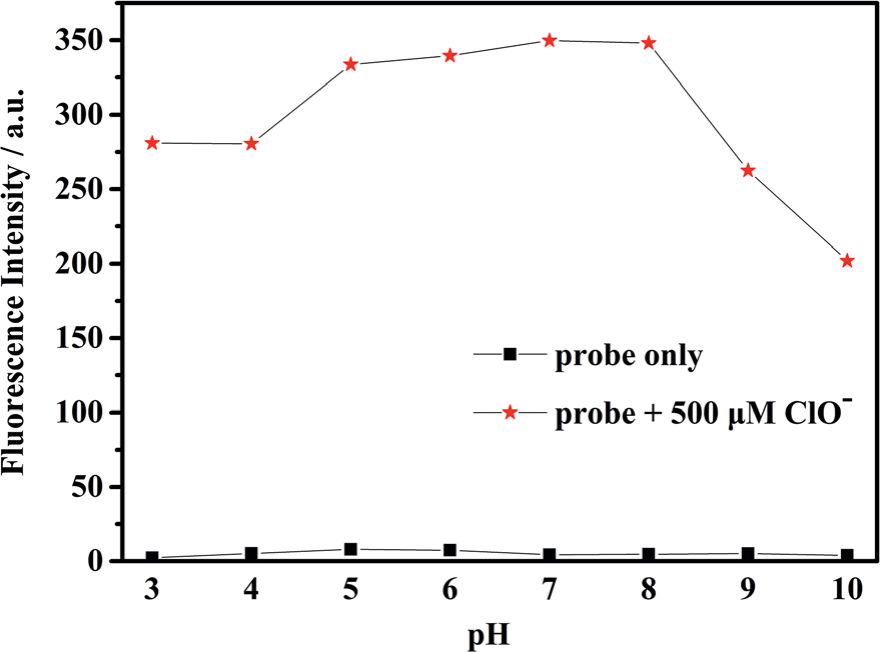
Fluorescence intensity of the probe mbac (10 μM) with and without ClO^−^ (500 μM) in water at different pH conditions, (*λ*_ex_ = 660 nm).

The fluorescence intensity of the probe at different pH levels is shown in Fig. 2. It can be seen that the probe is relatively stable in the pH range of 3–10, and the most suitable pH for measurement is in the range of 5–8. Therefore, we chose the pH 7.4 for future experiments.

In addition, in order to investigate the stability of the probe, we measured the fluorescence intensity of the probe at different temperatures. The results show that the probe is relatively stable; the specific data are provided in ESI (Fig S5, Tables S1 and S2†).

### Fluorescence spectral response of mbac

**Fig. 3:**
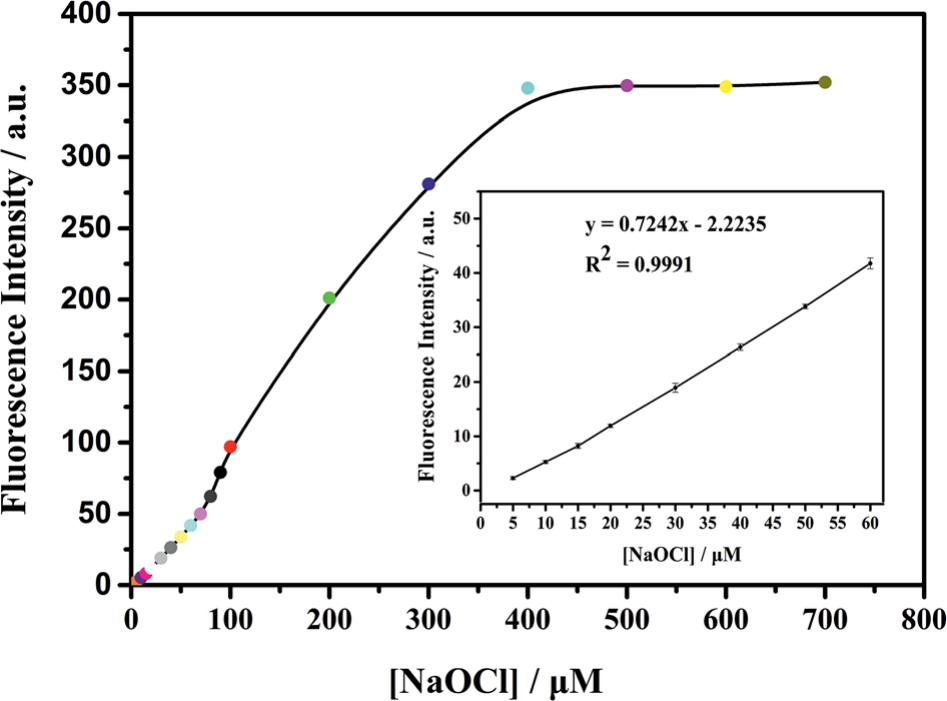
Fluorescence intensity increases with ClO^−^ concentration (*λ*_ex_ = 660 nm). The system contained the probe mbac (10 μM) in PBS (pH = 7.4). Each data point is the average of three parallel experiments.The inset graphic showing the correlation between the fluorescence response of mbac and the concentration of ClO^−^.

As shown in Fig. 3, the ClO^−^ concentration is linearly correlated with fluorescence intensity in the range of 0–60 μM, with a correlation coefficient of 0.9991 and detection limit as low as 0.1 μM. With the increase of fluorescence intensity (about 200 times), it is preliminarily proven that mbac could be used for *in vitro* detection of ClO^−^.

**Fig. 4:**
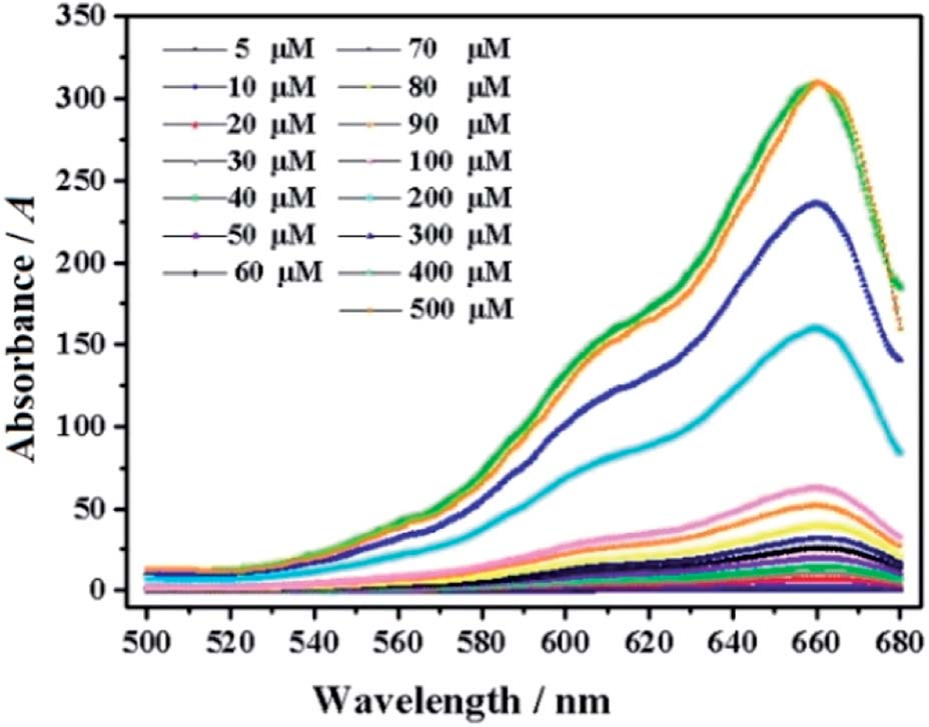
UV of probe mbac (10 μM) upon the addition of ClO^−^ (5–500 μM). The system was in PBS (pH = 7.4).

In order to further explore the *in vitro* detection ability of mbac, the UV response of the probe to various concentrations of ClO^−^ was studied. The UV-visible absorption spectra of the probe are shown in Fig. 4.

**Fig. 5:**
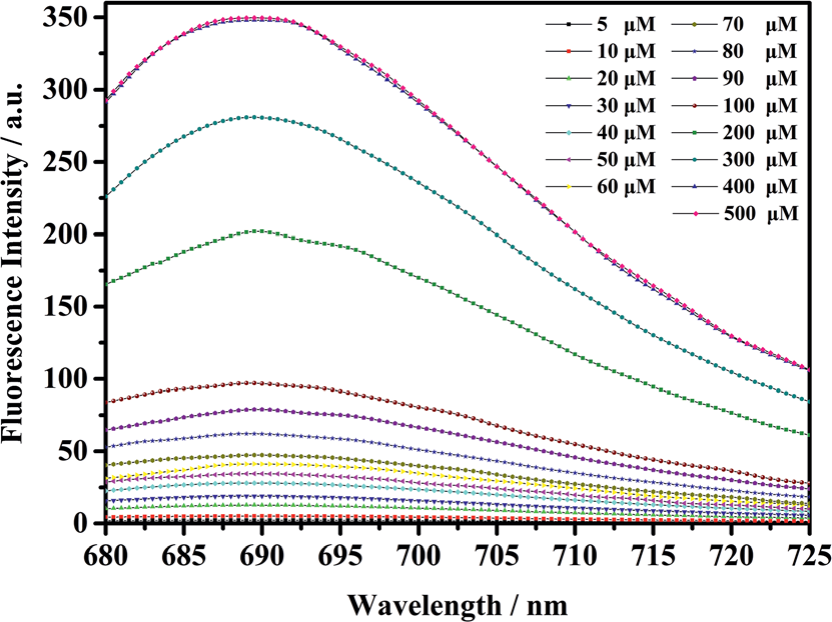
Fluorescence intensity increase of probe mbac (10 μM) upon the addition of ClO^−^ (5–500 μM), *λ*_ex_ = 660 nm. The system was PBS (pH = 7.4). The fluorescence intensity was determined at 690 nm.

As shown in Fig. 5, the fluorescent probe mbac has almost no emission in the visible region. After adding ClO^−^ solution, the fluorescence intensity peak appeared near 690 nm and reached the maximum after incubation at room temperature for 15 min, stabilizing within 30 minutes. As shown in Fig. 4 and 5, the fluorescence intensity at 690 nm was significantly enhanced with the addition of ClO^−^. From almost colorless to blue, the process of change is visible to the naked eye (Scheme 1).

The fluorescence excitation and emission wavelengths of mbac both reach the NIR range, which is very favorable for intracellular imaging. Fluorescence emission can be attributed to the cleavage of the amide bond to release the fluorescent group MB, which is confirmed by HPLC (Fig. S8†). The retention time of the probe was 12.87 minutes in HPLC, and for MB was 3.16 minutes. When ClO^−^ was added to mbac, the peaks of mbac disappeared, while the peak of MB appeared.

### Anti-interference

**Fig. 6:**
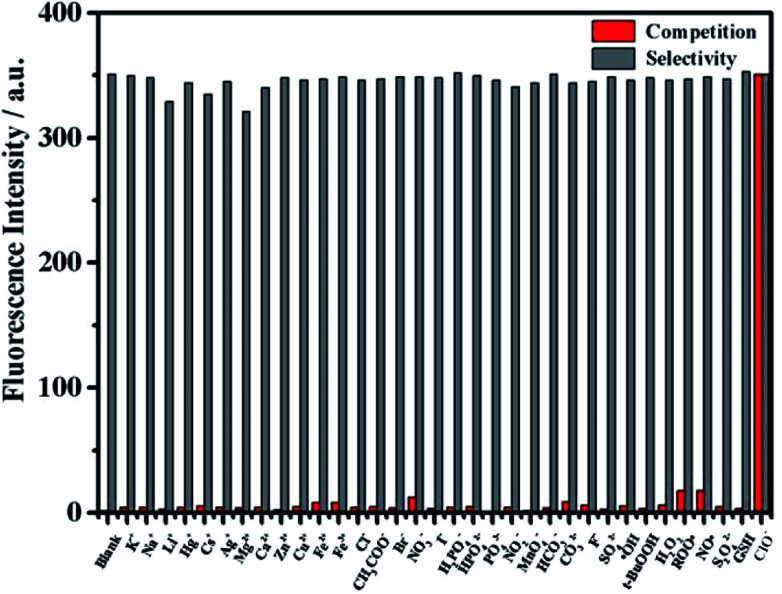
The fluorescence intensity (*λ*_ex_ = 660 nm) when the probe mbac (10 μM) was added to the solutions in PBS (pH = 7.4). The red bars indicate the fluorescence response of mbac to the interfering substance. K^+^, Na^+^, Li^+^, Hg^+^, Cs^+^, Ag^+^, Mg^2+^, Ca^2+^, Zn^2+^, Cu^2+^, Fe^2+^, Fe^3+^, Cl^−^, CH_3_COO^−^, Br^−^, NO_3_^−^, I^−^, H_2_PO_4_^−^, HPO_4_^2−^, PO_4_^3−^, NO_2_^−^, MnO_4_^−^, F^−^, SO_4_^2−^, HCO_3_^−^, CO_3_^2−^, ˙OH, *t*-BuOOH, H_2_O_2_, NO˙, S_2_O_4_^2−^, GSH and ClO^−^. Gray bars: after addition of 500 μM ClO^−^.

In order to verify the selectivity of the probe to ClO^−^, the fluorescence response of mbac was further studied after adding various interfering agents. Fluorescence intensity was measured for probe solutions containing 50 equivalents of various potential interfering ions. Then, 1 equivalent of ClO^−^ was added, and the intensity was measured again. The results show that the fluorescence intensity of mbac changed obviously only when ClO^−^ was added (see in Fig. 6).

In contrast, other ROS such as ˙OH, H_2_O_2_, *t*-BuOOH and NO˙; other ions such as K^+^, Na^+^, Li^+^, Hg^+^, Cs^+^, Ag^+^, Mg^2+^, Ca^2+^, Zn^2+^, Cu^2+^, Fe^2+^, Fe^3+^, Cl^−^, CH_3_COO^−^, Br^−^, NO_3_^−^, I^−^, H_2_PO_4_^−^, HPO_4_^2−^, PO_4_^3−^, NO_2_^−^, MnO_4_^−^, F^−^, SO_4_^2−^, HCO_3_^−^ and CO_3_^2−^; and other reducing agents such as S_2_O_4_^2−^ and GSH showed very weak fluorescence enhancement (Fig. 6), even at 50 equivalents. All of the results demonstrate that the probe mbac exhibits high selectivity to ClO^−^.

### Cytotoxicity assay

The basis of its application is safety, so it is necessary to study the probe's cytotoxicity. We selected LO2 cells (human normal liver cell) to measure the cytotoxicity of this probe. No apparent cytotoxicity was observed after LO2 cells were treated with mbac.

The methyl thiazolyl tetrazolium (MTT) assay was used to measure the cytotoxicity of mbac in LO2 cells. LO2 cells were seeded into a 96-well cell-culture plate. Cells were dosed with mbac at final concentrations ranging from 6.25 μM to 100 μM in each well of the plates. The cell survival was determined by measuring the absorbance at 490 nm by using a microplate reader. A calibration curve was prepared using SPSS to determine the IC50 of probe-mbac. Cytotoxicity at IC50 is the concentration of compound at which the optical density of treated cells (48 h) is reduced by 50% with respect to untreated cells using the MTT assay. The cell viability at various mbac doses was 95.2%, 100.5%, 106.5%, 102.1% and 104.8%. The results are shown in Fig. S9;†mbac exhibits a relatively low cytotoxicity in LO2 cells after exposure to up to 100 μM mbac for 48 h.

### Applications of mbac

**Fig. 7:**
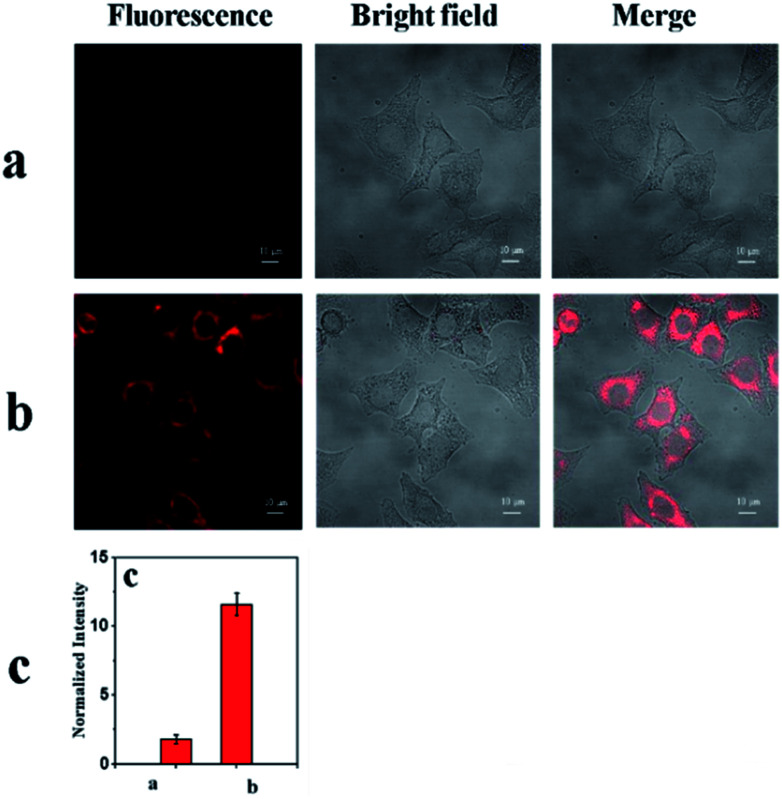
Fluorescence images of HepG2 cells. Left: fluorescence image; middle: bright-field image; right: merged image. (a) Cells were stained with 10 μM mbac (negative control) and incubated for 30 min at 20 °C. (b) The HepG2 cells were simultaneously incubated with 10 μM ClO^−^ and 10 μM probe for 30 min at 20 °C. (c) Relative fluorescence intensities of (a) and (b). The images were acquired using a confocal microscope with 633 nm excitation and 660–750 nm collection. Scale bar: 10 μm.

As shown in Fig. 7a, the probe mbac showed little fluorescence signal, consistent with fluorescence spectroscopy using HepG2 cells (human hepatocellular cancer cells).

On the other hand, a distinct emission response could be observed (Fig. 7b) after incubation with ClO^−^ for 30 min. The results show that the cell permeability of the probe mbac is good. Thus, mbac can be used to estimate the concentration of intracellular ClO^−^.

MBAC was also expected to be cell-membrane permeable, per the calculated log *P* of 3.82 obtained with the ALOGPS 2.1 programme.^25^ The predicted value of log *P* is within the range indicating good capacity to cross the plasma membrane.^26^

Under physiological conditions, ClO^−^ is highly reactive and short-lived; the average level of ClO^−^ generation from neutrophils is 0.47 nmol min^−1^ per 106 cells.^27^ Thus, when only the probe is added, the fluorescence in the cell is almost invisible. Furthermore, we hope to use this probe to detect ClO^−^ in living samples. The results are shown in Table 1; the fluorescence intensity of several samples is less than 10 a.u., tested in tap water andboiled water (Chaoyang, Beijing).

**Table 1:** Application of probe mbac (10 μM) in water samples (Beijing Chaoyang), compared with ClO^−^. Measured at *λ*_ex_ = 660 nm and measured at 690 nm

Samples	Average fluorescence intensity/a.u.	RSD
Tap water	8.305	0.084889
Boiled water	4.829	0.14549
ClO^−^	348.705	0.000875
Blank	2.185	0.009153

Furthermore, we found that boiled water contained less ClO^−^ than tap water. The results show that mbac is a common tool that can be successfully applied to the detection of ClO^−^ species in environmental samples.

## Conclusions

In conclusion, we have developed a fluorescent probe, mbac, whose absorption and emission wavelengths are both near infrared, with fast response and long response times, low cost and high specificity in the detection of ClO^−^. It is worth mentioning that the fluorescence intensity of the probe is enhanced by nearly 200 times, and the detection limit is 0.1 μM. The decomposition product has high safety as a clinical agent. In addition, we successfully measured the ClO^−^ concentration in different water samples by using mbac. The results of confocal laser microscopy demonstrate that the above theory can be applied at the cellular level. In summary, the probe mbac has a good prospect in measuring ClO^−^ in the fields of environment and biology.

## Conflicts of interest

There are no conflicts of interest to declare.

## Supplementary Material
